# Supplementary material for: Fear among Syrians: A Proposed Cutoff Score for the Arabic Fear of COVID-19 Scale
Source: PLoS One. 2022 Mar 11;17(3):e0264257. doi: 10.1371/journal.pone.0264257 (PMC8916619; doi:10.1371/journal.pone.0264257)
Supplement: S2 Table — (DOCX) [file pone.0264257.s002.docx]

**الملخص:**

أدّت جائحة فيروس كورونا (COVID-19) إلى مشاكل صحية نفسية من بينها الخوف، تتحقّق هذه الدّراسة من صحة النسخة العربيّة من مقياس الخوف من (COVID-19) وتقترح نقطة قطع جديدة لقياس الخوف من (COVID-19) بين السكان السورييّن، حيث قام ما مجموعه 3989 مشاركًا بملء استبيان عبر الإنترنت يتكون من معلومات اجتماعية وديموغرافية ، ومقياس الخوف من (COVID-19)، واستبيان صحة المريض (PHQ-9)، واضطراب القلق المعمم(GAD-7) ، تم استخدام تحليل خصائص تشغيل جهاز الاستقبال(ROC) لتحديد درجات القطع للخوف من مقياس (COVID-19) بالاستفادة من علاقته مع اضطراب القلق المعمم (GAD-7) واستبيان صحة المريض (PHQ-9)، كانت قيمة اختبار كرونباخ ألفا (Cronbach α) في مقياس الخوف العربي من (COVID-19) تساوي 0.896 ، مما يكشف عن استقرار جيد واتساق داخلي، كما كانت الارتباطات بين العناصر بين [0.420 - 0.868] والارتباطات المصححة الكلية كانت بين [0.614 - 0.768].
تم استنتاج نقطة قطع 17.5 من التحليل، ووفقًا لها صُنّفت 2111 (52.9٪) من الحالات على أنها حالات خوف شديد، يمكن استخدام نقطة القطع المستخلصة من هذه الدراسة لأغراض المسح لتحديد الأفراد الذين الأكثر عرضة لتطوير الخوف الشديد من (COVID-19)، ما يمكننا من تقديم تدابير وقائية وداعمة مبكرة.
