# Supplementary material for: Fear among Syrians: A Proposed Cutoff Score for the Arabic Fear of COVID-19 Scale
Source: PLoS One. 2022 Mar 11;17(3):e0264257. doi: 10.1371/journal.pone.0264257 (PMC8916619; doi:10.1371/journal.pone.0264257)
Supplement: S3 Table — (DOCX) [file pone.0264257.s003.docx]

**Supporting Information:**

**S2 Table. Arabic-version of the socio-demographic characteristics questions**

| الخصائص الاجتماعية والديموغرافية | |
| --- | --- |
| **العمر (سنوات):**  25-18 ⬜  34-26 ⬜  44-35 ⬜  ⬜-45 54  ⬜ فوق 55 | **الجنس:**  ⬜ ذكر  ⬜ أنثى |
| **الحالة العائلية:**  ⬜عازب/عازبة  ⬜متزوج/متزوجة  ⬜أخرى | **المستوى التعليمي:**  المدرسة الابتدائية**⬜**  المدرسة الإعدادية⬜  المدرسة الثانوية⬜  ⬜جامعة/كليّة  ⬜شهادة ماجستير  ⬜شهادة دكتوراه |
| **العمل:**  ⬜ رعاية الصحية  ⬜مؤسسة حكومية  ⬜ مؤسسة خاصة  ⬜ تجارة  ⬜ عسكري  ⬜ طالب  ⬜أخرى | **مكان الإقامة:**  ⬜ دمشق/ريف دمشق  ⬜ حماة  ⬜ حلب  ⬜ حمص  ⬜ طرطوس  ⬜ اللاذقية  ⬜ السويداء  ⬜ درعا  ⬜ الحسكة  ⬜ دير الزور  ⬜ ادلب  ⬜ الرقة  ⬜ القنيطرة |
| **المنطقة:**  ⬜ ريف  ⬜ مدينة | **الوضع المادي:**  ⬜ممتاز  جيد ⬜  متوسط ⬜  منخفض ⬜ |
| **عدد الأشخاص الذين تعيش معهم؟**  لوحدي ⬜  5-1⬜  5< ⬜ | |

**S3 Table. Arabic-version of FCV-19S**

| مقياس الخوف من كوفيد-19 | | | | | |
| --- | --- | --- | --- | --- | --- |
|  | **أوافق بشدة** | **أوافق** | **لا أوافق ولا أرفض (حيادي)** | **لا أوافق** | **لا أوافق بشدة** |
| **1. أنا خائف من فيروس كورونا-19 لأقصى حد** |  |  |  |  |  |
| **2. لتفكير بفيروس كورونا-19 يشعرني بعدم الارتياح؟** |  |  |  |  |  |
| **3. أشعر بتعرق في كفيّ عندما أفكر بفيروس كورونا-19** |  |  |  |  |  |
| **4. أخشى أن أفقد حياتي بسبب فيروس كورونا-19** |  |  |  |  |  |
| **5. ينتابني القلق أو التوتر عندما أشاهد أخباراً أو قصصاً عن فيروس كورونا-19** |  |  |  |  |  |
| **6. لا يمكنني النوم بسبب قلقي من الإصابة بعدوى فيروس كورونا-19** |  |  |  |  |  |
| **7. تتسارع دقات قلبي عندما أفكر بالإصابة بعدوى فيروس كورونا-19** |  |  |  |  |  |
