# Supplementary material for: Fear among Syrians: A Proposed Cutoff Score for the Arabic Fear of COVID-19 Scale
Source: PLoS One. 2022 Mar 11;17(3):e0264257. doi: 10.1371/journal.pone.0264257 (PMC8916619; doi:10.1371/journal.pone.0264257)
Supplement: Attachment — Submitted filename: PONE-D-21-14780R2.pdf [file pone.0264257.s004.pdf]

PONE-D-21-14780R2

by Anonymous Anonymous

Submission date: 10-Jul-2021 08:08AM (UTC+0400)
Submission ID: 1617774157
File name: PONE-D-21-14780_R2s.pdf (652.44K)
Word count: 6080
Character count: 31907

Manuscript

Click here to access/download;Manuscript;PLOS one AC #2.docx

Fear among Syrians: a Proposed Cutoff Score and Validation of the Arabic Fear of COVID-19 Scale

Authors: Fatema Mohsen[1*¶], Batoul Bakkar[1¶], Salma Khadem alsrouji[1&], Esraa Abbas[1&], Alma Najjar[1&], Marah Marrawi[2&], Youssef Latifeh[3,4&]

Affiliations:

1 Faculty of Medicine, Syrian Private University, Damascus, Syria

2 Department of Statistics, Syrian Private University, Damascus, Syria

3 Department of Psychiatry, Faculty of Medicine, Syrian Private University, Damascus, Syria

4 Professor, Department of Psychiatry Faculty of Medicine, Damascus University

Corresponding Author:

* Fatema Mohsen

Email: fatemamohsena@gmail.com

¶ These authors contributed equally to this work.

[&] These authors also contributed equally to this work.

Abstract:

COVID-19 pandemic has led to psychological health issues one of which is fear. This study validates the Arabic version of the fear of COVID-19 scale and suggests a new cutoff score to measure fear of COVID-19 among the Syrian Population. A total of 3989 participants filled an online survey consisting of socio-demographic information, the fear of COVID-19 scale, the patient health questionnaire 9-item, and the generalized anxiety disorder 7-item. Receiver operating characteristic analysis was used to define cutoff scores for the fear of COVID-19 scale in relation to generalized anxiety disorder 7-item and the patient health questionnaire 9-item. The Cronbach α value of the Arabic fear of COVID-19 scale was 0.896, revealing good stability and internal consistency. The inter-item correlations were between [0.420 – 0.868] and the corrected item-total correlations were between [0.614 – 0.768]. A cutoff point of 17.5 was deduced from analysis. According to the deduced cutoff point, 2111(52.9%) were classified as cases with extreme fear. This cutoff score deduced from this study can be used for screening purposes to identify individuals that may be most vulnerable towards developing extreme fear of COVID-19. Therefore, early preventive and supportive measures can then be delivered.

Keywords: Fear of COVID-19 scale; Anxiety; Depression; Cutoff; Syria; War.

Abbreviations: COVID-19: Coronavirus Disease 2019; WHO: World Health Organization; PHEIC: Public Health Emergency of International Concern; FCV-19S: Fear of COVID-19 Scale; PHQ-9: Patient Health Questionnaire 9-item; GAD-7: Generalized Anxiety Disorder 7-item; ROC: Receiver operating characteristic; IRB: Institutional Review Board; SPSS: Statistical Package for Social Sciences; SD: Standard Deviation; AUC: Area Under the Curve.

Introduction:

The novel Coronavirus disease-2019 (COVID-19) is a momentous threat to humanity's health that has emerged as an outbreak and was declared by the World Health Organization (WHO) as a Public Health Emergency of International Concern (PHEIC) (1). The first COVID-19 incident was noted in Wuhan, China in December 2019. This virus quickly crossed the borders, spreading all over the world, resulting in a never-ending pandemic burdened with the results of morbidity and mortality (2). There are live updates of latest cases, and deaths every second of the day, as well as official briefings, and prognostications about future cataclysmic events (3). Enumeration of all deaths, compared to the 0.28% crude mortality rate, which will continue to rise as more infections and deaths occur, produces an image of excess death, capturing both the direct burden of the pandemic and its indirect mortality burden (4, 5). 22 March 2020 marked the first officially reported COVID-19 case in Syria (6). The numbers have escalated since then with Syria now entering its third wave (7-9).

As the pandemic has embedded misconceptions around COVID-19, strict precautionary measures have been adopted by governments, such as physical distancing, self-isolation, and quarantine (10). These measures were implemented to curtail the tide of the pandemic in the

absence of vaccine or treatment at the time of the survey. During the Syrian lockdown, many streets have become ghost towns. Flights all over the globe have ceased. Business and medical conferences have been postponed to the unknown future. Mosques, museums, parks, schools, and universities have been closed and abandoned. Grocery and pharmacy stores are being cleared of over-the-counter medications, disinfectants, supplements, and herbal remedies. Households are stacked to the brim with groceries and toiletries, in preparation for a long draconian lockdown that may never end. National closures of businesses, public facilities and entertainment have resulted in less currency in circulation. All the above have shocked the economy drastically. The effects of the mass media and governments have had a toll on society, probably for several years to come; their disruptive actions are eliciting more damage to people around the globe than COVID-19. As a major health issue, the COVID-19 pandemic has triggered a multilevel global crisis, affecting individual's physical and psychological mental health. Thus, researchers are expressing concerns related to COVID-19 adverse effects on society's mental health and psychological well-being (11-23).

One of these psychological impacts is fear, a vital emotion necessary for an individual's survival by arousing adaptive defense responses against potentially threatening events or danger (24). The current threatening event is COVID-19, a negative emotional reaction to or persistent worry over contracting COVID-19, morbidity, and mortality (25). Added to the previous are changes in our daily lives working from home, temporary unemployment, home-schooling, and lack of socializing. The fear is likely to become a more pressing concern, as the pandemic sustains high infection, morbidity, and mortality rates. These concerns have pushed researchers to assess the fear of COVID-19 in different regions of the world and identify the possible triggers (26).

Fear may lead to the incidence of new mental health disorders or worsening pre-existing psychiatric symptoms such as depression, anxiety, and post-traumatic stress disorder, and even suicide (13, 27, 28). A recent study has developed the Fear of COVID-19 Scale (FCV-19S) to complement the clinical efforts in preventing the spread and treating of COVID-19 cases (29). The psychometric evidence of the FCV-19S demonstrated satisfactory results (30-36). A few studies thereafter have validated the questionnaire in various languages and examined associations with socio-demographic characteristics (34, 37-43). However, only one study as of yet has proposed a cutoff score for the FCV-19S (44). Given that Syria is one of the most vulnerable countries in the world to be tormented by mental health disorders due to both COVID-19 and war. This study aimed to assess the fear of COVID-19 among the Syrian population, validate the FCV-19S, and identify an appropriate cutoff score to differentiate adults with extreme fear of COVID-19 from those with a normal fear reaction.

Materials and methods:

Study design, setting, and participants:

This web-based cross-sectional study was conducted using an Arabic questionnaire over a period of 12 days between May 2 and May 14 of 2020. All participants aged 18 and above, residing in Syria, who completed the survey, were included in the study. A convenience sampling method was used in the study. The questionnaire was distributed through various social media platforms. After providing informed consent online, participants were directed to the first part of the survey to complete questions about socio-demographic information including gender, age, residence, education level, occupation, and economic status. Both the Arabic and English

version of the socio-demographic questions are provided in S1 and S2 Tables. Participants were also asked about the history of chronic diseases. To validate the Arabic version of the FCV-19S, a dataset comprising 3989 participants was used

Adaptation of FCV-19S into Arabic:

The original FCV-19S was translated into Arabic using a forward-backward translation technique (29). First, the FCV-19S was translated into Arabic by two medical translators who are fluent in both Arabic and English. The authors, who are fluent in Arabic and English, reviewed the provisional Arabic translation and no anomalies were identified with the translation. Third, the approved Arabic translation draft of the scale was then translated back into English by another medical translator who was unacquainted with the original English FCV-19S. Both the forward and backward translations of the scale were then compared for equivalence and checked for cultural appropriateness among the authors. The approved Arabic questionnaire was piloted with 20 volunteers to assess the scale's reliability, clarity, relevance, and acceptability of the survey. These volunteers were excluded from the final sample and statistical analysis to avoid bias. Based on the feedback no modifications were required. The Arabic version of the FCV-19S is provided in S3 Table.

Measures:

The FCV-19S (after translation) was used to evaluate the symptoms of fear . The FCV-19S is a one-dimensional scale that measures one's fear level of COVID-19 it consists of 7 items and is scored on a 5-point scale, ranging from 1 (strongly disagree) to 5 (strongly agree). A total score is calculated by summing all item scores with a possible total score ranging between 7 and 35. Higher scores indicate greater levels of fear of COVID-19 (42).

The Arabic Patient Health Questionnaire 9-item (PHQ-9) was used in the study to assess depression symptom severity (45). Items on the PHQ-9 were rated on a 4-point Likert scale: 0 = not at all, 1 = several days, 2 = more than half the days, and 3 = nearly every day), providing a 0-27 severity score range. The scores were categorized into 5 groups: none (0–4), mild (5–9), moderate (10–14), moderately severe (15-19), and severe (20–27).

The Arabic Generalized Anxiety Disorder 7-item (GAD-7) was used in this study to assess anxiety symptom severity.(46) Items on the GAD-7 were rated on a 4-point Likert scale: 0 = not at all, 1 = several days, 2 = more than half the days, 3 = nearly every day, providing a 0-21 severity score range. The Gad-7 is a self-rated scale used to evaluate the severity of the 4 most common anxiety disorders (Generalized Anxiety Disorder, Panic Disorder, Social Phobia, and Post Traumatic Stress Disorder), cutoff score of 0–4 indicates no anxiety symptoms. The scores were categorized into 4 groups: none (0-4), mild (5-9), moderate (10-14), and severe (15-21) (47).

Ethics:

The study was ethically approved by the Institutional Review Board (IRB) of the Faculty of Medicine, Syrian Private University. The study was not granted a registered number.

Statistical analysis:

Data analysis was conducted using the Statistical Package for Social Sciences version 25.0 (SPSS Inc., Chicago, IL, United States). Descriptive analysis, including frequencies, percentages, means, and standard deviations (SD) were applied. The Cronbach's α test and inter-item correlation were used to assess the internal consistency, with satisfactory reliability set at ≥

0.70 and between 0.20 and 0.40 respectively (48, 49). The corrected item-total correlation was used to assess the coherence of the FCV-19S (values > 0.4 are acceptable). The current analysis focuses on the association between the FCV-19S scale and other variables relevant to the participants' psychological reaction to COVID-19, including anxiety, and depression. Receiver operating characteristic (ROC) analysis was used to define cutoff scores for the FCV-19S scale in relation to GAD-7 and PHQ-9 serving as external criteria. The Youden-Index was used to determine the optimal cutoff score and to reduce the risk of misclassification. A dichotomous variable was created out of the total for both GAD-7 and PHQ-9 using the cutoff point of 10 to assess anxiety and depressive symptoms respectively (45, 47). After identifying the cutoff points, participants with a sum-score above the given cutoff value were considered cases with extreme fear. Those below the deduced cutoff value were regarded as non-fear cases.

Results:

Socio-demographic characteristics of participants

Of 5000 total participants invited to take part in the study, 4,430 gave informed consent. A final sample size of 3989 participants (response rate = 79.8%) met the inclusion criteria for the study. The 441 participants who did not meet the inclusion criteria were either below 18, residing outside Syria, or uncompleted survey. Most participants were female 2935 (73.5%), single 3096 (77.6%), students 2397 (60.1%), and residing in Damascus 1412 (35.4%). Participant ages ranged from 18 to 70 years, with the age group 18-25 years representing a majority 2870 (71.9%). A total of 416 (10.4%) and 1522 (38.1%) participants stated they had poor and

moderate economic status respectively. 556 (15.9%) mentioned a history of chronic diseases Table 1.

Table 1. Socio-demographic characteristics of participants

**Gender (%)**	**Male**	1054 (26.4)
	**Female**	2935 (73.5)
**Age (%)**	**18-25**	2870 (71.9)
	**26-34**	685 (17.2)
	**35-44**	261(6.5)
	**45-54**	121 (3.0)
	**55 <**	52 (1.4)
**Social Status (%)**	**Single**	3096 (77.6)
	**Married**	714 (17.9)
	**Other**	179 (4.5)
**Economic Status (%)**	[1]**Excellent**	251 (6.3)

	[2]Good	1800 (45.1)
	[3]Moderate	1522 (38.1)
	[4]Poor	416 (10.4)
**Chronic disease(s) (%)**	**Yes**	556 (13.9)
	**No**	3433 (86)
**Education (%)**	**Primary School**	21 (0.5)
	**Intermediate School**	115 (2.9)
	**Secondary school**	370 (9.3)
	**College/ University**	3271 (82)
	**Master's degree**	185 (4.6)
	**PhD**	27 (0.7)
**Occupation (%)**	**Health care worker**	259 (6.5)
	**Government institution**	239 (6.0)

	**Private institution**	202 (5.1)
	**Business**	202 (5.1)
	**Military**	35 (0.9)
	**Student**	2397 (60.1)
	**Other**	655 (16.4)
**Household members (%)**	**Alone**	54 (1.4)
	**1-5**	2474 (62)
	**>5**	1461 (36.6)

[1]Poor: income does not provide essential needs for the family. [1]Excellent: income provides luxury requirements. [2]Good: income provides essential needs and some luxury requirements. [3]Moderate: income provides essential needs for the family but no more. [4]Poor: income does not provide essential needs for the family.

Validity and Cutoff Score analysis:

The FCV-19S scores ranged between 7 and 35, the mode score was 14, and the mean score was 18.5 (± 6.009) Fig 1. Participants' responses to the FCV-19S are shown in Table 2. The Cronbach α value of the Arabic FCV-19S was 0.896, revealing good stability and internal consistency. The inter-item correlations were between [0.420 – 0.868] and the corrected item-total correlations were between [0.614 – 0.768].

The responses to the GAD-7 scale were classified into two groups: non-anxious symptoms (score < 9) and anxious symptoms (9 < score ≤ 21). The responses to the PHQ-9 scale were classified into two groups: non-depressed symptoms (score < 9) and depressed symptoms (9 < score ≤ 27) Table 3.

ROC analysis was used to evaluate the efficacy of the FCV-19S in predicting anxiety and depression factors. The proposed cutoff score was determined by the optimal sensitivity and specificity level, Table 4, revealing the following:

- Unsatisfactory predictive power for the FCV-19S in disclosure of anxiety symptoms assessed by GAD-7 was found. The best cutoff was at 18.5, with a sensitivity of 53% and specificity of 57.5%, as the area under the curve (AUC) was 0.560 with a statistical significance (p-value < 0.001), a confidence interval of 95% CI=[0.541, 0.578] Table 4 and Fig 2.
- Unsatisfactory predictive power for the FCV-19S in disclosure of depressive symptoms assessed by PHQ-9 was found. The best cutoff was at 17.5 with a sensitivity of 55.1% and specificity of 49.6%, AUC=0.520 was statistically significant (p-value < 0.001), a confidence interval of 95% CI= [0.502, 0.538] Table 4 and Fig 3.

A cutoff point of 17.5 was deduced from the anxiety and depression ROC analysis. According to the deduced cutoff point, the majority 2111(52.9%) were classified as cases with extreme fear Table 3.

Table 2. . Participants response to FCV-19S

	**Response to FCV-19S**				
**FCV-19S**	**Totally disagree**	**Disagree (%)**	**Neutral (%)**	**Agree (%)**	**Totally Agree (%)**

	(%)				
**1-I am often afraid of the emerging COVID-19**	261 (6.5)	826 (20.7)	1557 (39.0)	951 (23.8)	394 (9.9)
**2- I feel uncomfortable when I think about the new COVID-19**	279 (7.0)	906 (22.7)	1115 (28.0)	1228 (30.8)	461 (11.6)
**3- My hands get sweaty when I think about the new COVID-19**	962 (24.1)	1983 (49.7)	719 (18.0)	119 (3.0)	206 (5.2)
**4- I am afraid of dying due to infection with the emerging COVID-19**	685 (17.2)	1579 (39.6)	892 (22.4)	463 (11.6)	370 (9.3)
**5- I feel anxious and nervous when I follow news or posts about COVID-19 on social media**	390 (9.8)	968 (24.3)	1002 (25.1)	1070 (26.8)	559 (14.0)
**6- I cannot sleep because of my anxiety about infection with the emerging COVID-19**	1045 (26.2)	1875 (47.0)	688 (17.2)	152 (3.8)	229 (5.7)
**7- I feel my heart racing and palpitations when I think about the emerging COVID-19**	1023 (25.6)	1843 (46.2)	670 (16.8)	211 (5.3)	242 (6.1)

Table 3. Classification of participants according to FCV-19S, GAD-7, and PHQ-9 cutoff points

	Yes (%)	No (%)
**FCV-19S**	211 (52.9)	1878 (47.1)
**GAD-7**	1484 (37.2)	2505 (62.8)
**PHQ-9**	2176 (54.6)	1813 (45.4)

Table 4. Prediction validity of the FCV-19S

	**FCV-19S Score**	**Sensitivity**	**Specificity**	**AUC**	**CI (95%)**	**p-value**	**Youden's Index**
**Anxiety**	18.5	0.532	0.575	0.560	[0.541 , 0.578]	<0.001	0.107
**Depression**	17.5	0.551	0.496	0.520	[0.502 , 0.538]	<0.001	0.047

Discussion:

The current study was performed to validate the Arabic version of the FCV-19S and propose a cutoff to differentiate those with extreme fear from those with normal fear reactions towards COVID-19. Obtaining a cutoff is a pivotal and common practice in the psychiatry field to differentiate those into either cases or non-cases (50-54). Identifying cases and non-cases with extreme fear will allow us to stratify those in need for further assessment by a specialist in psychiatry. Once assessed, appropriate management can be given if indicated. Although many studies have used the FCV-19S to assess fear among various populations, without a cutoff score

we cannot further analyze the results and compare and interpret findings with different populations (55).

Our study revealed a good internal consistency (Cronbach's α = 0.896). Our finding was in line with other studies with a range of 0.82 to 0.88 (29, 43, 56, 57). The inter-item correlations were between [0.420 – 0.868] and the corrected item-total correlations were between [0.614 – 0.768], whereas it was [0.35 - 0.66] and [0.57- 0.74] respectively in a Saudi-Arabian study (58).

ROC curves were constructed to determine the FCV-19S cutoff point. A cutoff point of 17.5 was deduced, participants scoring ≥ 17.5 were classified as having extreme fear of COVID-19, whereas participants scoring below this threshold were classified as having normal fear of COVID-19. Only one study has defined a cutoff for the FCV-19S, with a threshold of 16.5 and higher (44).

This study revealed a weak discriminatory ability from the AUC outcomes, whereas its accuracy was moderate in a Greek study (59). The low precision of AUC signifies that not all individuals with depression or anxiety symptoms have an unhealthy fear of COVID-19 and vice versa. This may be attributed to over a decade of conflict and the implications it induced on the mental health of the Syrian population (60). Thus, Syrians have countless factors contributing to their depressive and anxiety symptoms unlike the majority of the globe. Also, the FCV-19S was designed to assess fear towards COVID-19 while the GAD-7 and PHQ-9 are not aimed at assessing depression and anxiety towards the COVID-19 pandemic.

Fear of COVID-19 may be a factor that impacts an individual's protective behaviors, including vaccination uptake (61). Therefore, one should have a certain level of fear but not extreme fear as individuals lack perception to respond to threats faced (62). Our study revealed a

prevalence of 52.9% of Syrians was classified as having extreme fear towards COVID-19. The prevalence of extreme fear among Syrians was higher in comparison with the Greeks (40.3%), despite their lower cutoff. The higher prevalence among Syrians can be attributed to the devastating impacts of war, driving the country into a prolonged economic recession. The devastating conflict has resulted in a Syrian healthcare system that is severely under-equipped and lacks the capacity to contain such a pandemic. With many healthcare professionals fleeing the country, a low number of ventilators, and many hospitals bombarded to rubble, Syrians must feel constant fear for their lives (63).

All the above factors have exacted immense effects on the mental health of Syrians. The Syrian ministry of health provides 3 hospitals for substance abuse and mental illness: Ibn Rushd Hospital in Damascus, Ibn Sina Hospital in Rural Damascus, and Ibn Khaldoun Hospital in Aleppo. Ibn Khaldoun Hospital was bombarded on 25/12/2012, leaving only 2 working hospitals. Our study showed that 54.6% and 37.2% of Syrians are suffering from moderate to severe depressive and anxiety symptoms respectively. Grievously, 90% of cases are left unattended, untreated, and unmanaged, as only 80 psychiatrists are available in Syrian territories (64, 65). We must call on national and international organizations to aid us Syrians, reconstruct mental health services, and assist in providing skilled healthcare workers for the suffering people of Syria.

Limitations:

The present study has several limitations in its design. First, the convenience sampling used in this study may have limited results' generalizability due to a "volunteer-effect" and the potential underrepresentation of less educated and socially disadvantaged participants. Second, credible published national data regarding the socio-demographic variables of Syrians are not available to evaluate the representativeness of our sample. Third, the cross-sectional design limited the assessment of prospective effects of fear over time. Fourth, the proposed cutoff aimed to further evaluate the scale's predicting ability to screen for cases with extreme fear, and cannot be used for diagnostic purposes. Fifth, a lack of other instruments assessing the same construct during the conduct of the study such as health anxiety and posttraumatic stress disorder. Sixth, the cutoff should ideally only be used on Syrian populations due to the characteristics of the participants in the study. Therefore, the proposed cutoff point must be interpreted with particular caution.

Conclusion:

This study provided empirical support for the scale's adequacy to assess COVID-19-related fear and determined a cutoff point of ≥ 17.5 with unsatisfactory predictive power for anxiety and depression. This cutoff score can be used for screening purposes to identify individuals that may be most vulnerable towards developing psychiatric symptomatology. Therefore, require further assessment to identify high-risk individuals and deliver early preventive and/or supportive measures. However, the Syrian healthcare system is under constant strain due to the effects of war and the COVID-19 pandemic, and such measures may only exist in our imaginations.

Supporting Information:

S1 Table. English-version of the socio-demographic characteristics questions

S2 Table. Arabic-version of the socio-demographic characteristics questions

S3 Table. Arabic-version of FCV-19S

6

Acknowledgments:

We are thankful to the management of the Syrian Private University for the support in the field of medical training and research. We are thankful to everyone who participated in this study.

References:

1. WHO. Getting your workplace ready for COVID-19: how COVID-19 spreads, 19 March 2020. World Health Organization: World Health Organization; 2020.
2. WHO. WHO Coronavirus Disease (COVID-19) Dashboard: World Health Organization; 2020 [Available from: https://covid19.who.int/.
3. Worldometer. COVID-19 CORONAVIRUS PANDEMIC [Available from: https://www.worldometers.info/coronavirus/.
4. Setel P, AbouZahr C, Atuheire EB, Bratschi M, Cercone E, Chinganya O, et al. Mortality surveillance during the COVID-19 pandemic. Bulletin of the World Health Organization. 2020;98(6):374.
5. Coronavirus (COVID-19) Mortality Rate [Internet]. Available from: https://www.worldometers.info/coronavirus/coronavirus-death-rate/.
6. McKernan B. Syria con rms rst Covid-19 case amid fears of catastrophic spread. The Guardian. 2020.
7. Al-awsat A. Syria Grapples with Third COVID-19 Wave. Alsharq Al-awsat Tuesday, 2 March, 2021 - 08:15.
8. Tripp JC, McDevitt-Murphy ME, Avery ML, Bracken KLJJodd. PTSD symptoms, emotion dysregulation, and alcohol-related consequences among college students with a trauma history. 2015;11(2):107-17.
9. WHO. COVID-19 Humanitarian Update No. 23: World Health Organization; 2021 [Available from: https://reliefweb.int/report/syrian-arab-republic/syrian-arab-republic-covid-19-humanitarian-update-no-23-1-february-2021.

10. WHO. Coronavirus ,Overview ,Prevention and Symptoms: World Health Organization; 2020 [Available from: https://www.who.int/health-topics/coronavirus#tab=tab_2.
11. Rajkumar RP. COVID-19 and mental health: A review of the existing literature. Asian journal of psychiatry. 2020;52:102066.
12. Holmes EA, O'Connor RC, Perry VH, Tracey I, Wessely S, Arseneault L, et al. Multidisciplinary research priorities for the COVID-19 pandemic: a call for action for mental health science. The Lancet Psychiatry. 2020.
13. Ornell F, Schuch JB, Sordi AO, Kessler FHP. "Pandemic fear" and COVID-19: mental health burden and strategies. Brazilian Journal of Psychiatry. 2020;42(3):232-5.
14. Fung XC, Siu AM, Potenza MN, O'brien KS, Latner JD, Chen C-Y, et al. Problematic use of internet-related activities and perceived weight stigma in schoolchildren: A longitudinal study across different epidemic periods of COVID-19 in China. 2021;12:700.
15. Malik S, Ullah I, Irfan M, Ahorsu DK, Lin C-Y, Pakpour AH, et al. Fear of COVID-19 and workplace phobia among Pakistani doctors: A survey study. 2021;21(1):1-9.
16. Mahmoudi H, Saffari M, Movahedi M, Sanaeinasab H, Rashidi-Jahan H, Pourgholami M, et al. A mediating role for mental health in associations between COVID-19-related self-stigma, PTSD, quality of life, and insomnia among patients recovered from COVID-19. 2021;11(5):e02138.
17. Chen I-H, Chen C-Y, Pakpour AH, Griffiths MD, Lin C-Y, Li X-D, et al. Problematic internet-related behaviors mediate the associations between levels of internet engagement and distress among schoolchildren during COVID-19 lockdown: A longitudinal structural equation modeling study. 2021;10(1):135-48.
18. Pramukti I, Strong C, Sitthimongkol Y, Setiawan A, Pandin MGR, Yen C-F, et al. Anxiety and Suicidal Thoughts During the COVID-19 Pandemic: Cross-Country Comparative Study Among Indonesian, Taiwanese, and Thai University Students. 2020;22(12):e24487.
19. Chen C-Y, Chen I-H, O'Brien KS, Latner JD, Lin C-YJIJoO. Psychological distress and internet-related behaviors between schoolchildren with and without overweight during the COVID-19 outbreak. 2021;45(3):677-86.
20. Ahorsu DK, Lin C-Y, Pakpour AHJG, Medicine G. The Association Between Health Status and Insomnia, Mental Health, and Preventive Behaviors: The Mediating Role of Fear of COVID-19. 2020;6:2333721420966081.
21. Chang K-C, Strong C, Pakpour AH, Griffiths MD, Lin C-YJJotFMA. Factors related to preventive COVID-19 infection behaviors among people with mental illness. 2020;119(12):1772-80.
22. Lin C-Y, Broström A, Griffiths MD, Pakpour AHJIi. Investigating mediated effects of fear of COVID-19 and COVID-19 misunderstanding in the association between problematic social media use, psychological distress, and insomnia. 2020;21:100345.
23. Chen I-H, Chen C-Y, Pakpour AH, Griffiths MD, Lin C-YJJotAAoC, Psychiatry A. Internet-related behaviors and psychological distress among schoolchildren during COVID-19 school suspension. 2020;59(10):1099.
24. Hamm AO. Fear, anxiety, and their disorders from the perspective of psychophysiology. Psychophysiology. 2020;57(2):e13474.
25. Van Bavel JJ, Baicker K, Boggio PS, Capraro V, Cichocka A, Cikara M, et al. Using social and behavioural science to support COVID-19 pandemic response. Nature human behaviour. 2020;4(5):460-71.
26. Ahorsu DK, Lin C-Y, Imani V, Saffari M, Griffiths MD, Pakpour AH. The Fear of COVID-19 Scale: Development and Initial Validation. International Journal of Mental Health and Addiction. 2020.
27. Gunnell D, Appleby L, Arensman E, Hawton K, John A, Kapur N, et al. Suicide risk and prevention during the COVID-19 pandemic. The Lancet Psychiatry. 2020;7(6):468-71.

28. Tso IF, Park S. Alarming levels of psychiatric symptoms and the role of loneliness during the COVID-19 epidemic: A case study of Hong Kong. Psychiatry research. 2020;293:113423.
29. Ahorsu DK, Lin C-Y, Imani V, Saffari M, Griffiths MD, Pakpour AH. The fear of COVID-19 scale: development and initial validation. International journal of mental health and addiction. 2020:1-9.
30. Chang K-C, Hou W-L, Pakpour AH, Lin C-Y, Griffiths MDJIJoMH, Addiction. Psychometric testing of three COVID-19-related scales among people with mental illness. 2020:1-13.
31. Pakpour AH, Griffiths MD, Chang K-C, Chen Y-P, Kuo Y-J, Lin C-YJB, behavior,, et al. Assessing the fear of COVID-19 among different populations: A response to Ransing et al.(2020). 2020;89:524-5.
32. Andrade EF, Pereira LJ, Oliveira APLd, Orlando DR, Alves DAG, Guilarducci JdS, et al. Perceived fear of COVID-19 infection according to sex, age and occupational risk using the Brazilian version of the Fear of COVID-19 Scale. 2020:1-10.
33. Haktanir A, Seki T, Dilmaç BJDS. Adaptation and evaluation of Turkish version of the fear of COVID-19 scale. 2020:1-9.
34. Caycho-Rodríguez T, Vilca LW, Cervigni M, Gallegos M, Martino P, Portillo N, et al. Fear of COVID-19 scale: Validity, reliability and factorial invariance in Argentina's general population. Death Studies. 2020:1-10.
35. Soares FR, Afonso RM, Martins AP, Pakpour AH, Rosa CPJDS. The fear of the COVID-19 Scale: validation in the Portuguese general population. 2021:1-7.
36. Moreta-Herrera R, López-Calle C, Caycho-Rodríguez T, Cabezas Guerra C, Gallegos M, Cervigni M, et al. Is it possible to find a bifactor structure in the Fear of COVID-19 Scale (FCV-19S)? Psychometric evidence in an Ecuadorian sample. 2021:1-11.
37. Giordani RCF, Zanoni da Silva M, Muhl C, Giolo SR. Fear of COVID-19 scale: Assessing fear of the coronavirus pandemic in Brazil. Journal of Health Psychology. 2020:1359105320982035.
38. Midorikawa H, Aiba M, Lebowitz A, Taguchi T, Shiratori Y, Ogawa T, et al. Confirming validity of The Fear of COVID-19 Scale in Japanese with a nationwide large-scale sample. PloS one. 2021;16(2):e0246840.
39. Elemo AS, Satici SA, Griffiths MD. The Fear of COVID-19 Scale: Psychometric Properties of the Ethiopian Amharic Version. International Journal of Mental Health and Addiction. 2020:1-12.
40. Mertens G, Gerritsen L, Duijndam S, Salemink E, Engelhard IM. Fear of the coronavirus (COVID-19): Predictors in an online study conducted in March 2020. Journal of Anxiety Disorders. 2020;74:102258.
41. Lin CY, Hou WL, Mamun MA, Aparecido da Silva J, Broche-Pérez Y, Ullah I, et al. Fear of COVID-19 Scale (FCV-19S) across countries: Measurement invariance issues. Nursing Open. 2021.
42. Alyami M, Henning M, Krägeloh CU, Alyami H. Psychometric evaluation of the Arabic version of the Fear of COVID-19 Scale. International journal of mental health and addiction. 2020:1-14.
43. Satici B, Gocet-Tekin E, Deniz ME, Satici SA. Adaptation of the Fear of COVID-19 Scale: Its association with psychological distress and life satisfaction in Turkey. International Journal of Mental Health and Addiction. 2020:1-9.
44. Nikopoulou V, Holeva V, Parlapani E, Karamouzi P, Voitsidis P, Porfyri G, et al. Mental Health Screening for COVID-19: a Proposed Cutoff Score for the Greek Version of the Fear of COVID-19 Scale (FCV-19S). International Journal of Mental Health and Addiction. 2020:1-14.
45. Sawaya H, Atoui M, Hamadeh A, Zeinoun P, Nahas Z. Adaptation and initial validation of the Patient Health Questionnaire–9 (PHQ-9) and the Generalized Anxiety Disorder–7

Questionnaire (GAD-7) in an Arabic speaking Lebanese psychiatric outpatient sample. Psychiatry research. 2016;239:245-52.
46. AlHadi AN, AlAteeq DA, Al-Sharif E, Bawazeer HM, Alanazi H, AlShomrani AT, et al. An arabic translation, reliability, and validation of Patient Health Questionnaire in a Saudi sample. Annals of general psychiatry. 2017;16(1):1-9.
47. Löwe B, Decker O, Müller S, Brähler E, Schellberg D, Herzog W, et al. Validation and standardization of the Generalized Anxiety Disorder Screener (GAD-7) in the general population. Medical care. 2008:266-74.
48. Cronbach LJ, Shavelson RJ. My current thoughts on coefficient alpha and successor procedures. Educational and psychological measurement. 2004;64(3):391-418.
49. Piedmont RL. Inter-item Correlations. In: Michalos AC, editor. Encyclopedia of Quality of Life and Well-Being Research. Dordrecht: Springer Netherlands; 2014. p. 3303-4.
50. Blanco V, Guisande MA, Sánchez MT, Otero P, Vázquez FL. Spanish validation of the 10-item Connor–Davidson Resilience Scale (CD-RISC 10) with non-professional caregivers. Aging & mental health. 2019;23(2):183-8.
51. Ebrahimi A, Rief W, Mirshahzadeh P, Zanjani HA, Dehsorkhi HN, Roohafza H, et al. Psychometric properties of the Persian version of screening for Somatic Symptom Disorders-7 (SOMS-7). Iranian journal of psychiatry. 2018;13(4):264.
52. Lee SA. Measuring coronaphobia: the psychological basis of the Coronavirus Anxiety Scale. Dusunen Adam. 2020;33(2):107-8.
53. Oliveira MA, Bendo CB, Paiva SM, Vale MP, Serra-Negra JM. Determining cut-off points for the dental fear survey. The Scientific World Journal. 2015;2015.
54. Silva TBF, Osório FL, Loureiro SR. SDQ: discriminative validity and diagnostic potential. Frontiers in Psychology. 2015;6(811).
55. Cook DA, Beckman TJ. Current concepts in validity and reliability for psychometric instruments: theory and application. The American journal of medicine. 2006;119(2):166. e7-. e16.
56. Soraci P, Ferrari A, Abbiati FA, Del Fante E, De Pace R, Urso A, et al. Validation and Psychometric Evaluation of the Italian Version of the Fear of COVID-19 Scale. International Journal of Mental Health and Addiction. 2020.
57. Satici B, Gocet-Tekin E, Deniz ME, Satici SA. Adaptation of the Fear of COVID-19 Scale: Its Association with Psychological Distress and Life Satisfaction in Turkey. International Journal of Mental Health and Addiction. 2020.
58. Alyami M, Henning M, Krägeloh CU, Alyami H. Psychometric Evaluation of the Arabic Version of the Fear of COVID-19 Scale. International Journal of Mental Health and Addiction. 2020.
59. Mandrekar JN. Receiver operating characteristic curve in diagnostic test assessment. Journal of Thoracic Oncology. 2010;5(9):1315-6.
60. Kakaje A, Al Zohbi R, Hosam Aldeen O, Makki L, Alyousbashi A, Alhaffar MBA. Mental disorder and PTSD in Syria during wartime: a nationwide crisis. BMC Psychiatry. 2021;21(1):2.
61. Wang P-W, Ahorsu DK, Lin C-Y, Chen I-H, Yen C-F, Kuo Y-J, et al. Motivation to have covid-19 vaccination explained using an extended protection motivation theory among university students in china: The role of information sources. 2021;9(4):380.
62. Rippetoe PA, Rogers RWJJop, psychology s. Effects of components of protection-motivation theory on adaptive and maladaptive coping with a health threat. 1987;52(3):596.
63. Gharibah M, Mehchy Z. COVID-19 pandemic: Syria's response and healthcare capacity. 2020.
64. Hedar M. Mental health during the Syrian crisis: How Syrians are dealing with the psychological effects. Int'l Rev Red Cross. 2017;99:927.
65. Abou-Saleh M, Mobayed M. Mental health in Syria. International Psychiatry. 2013;10(3):58-60.

PONE-D-21-14780R2

ORIGINALITY REPORT

34%	22%	33%	5%
SIMILARITY INDEX	INTERNET SOURCES	PUBLICATIONS	STUDENT PAPERS

PRIMARY SOURCES

1	V. A. Nikopoulou, V. Holeva, Eleni Parlapani, P. Karamouzi et al. "Mental Health Screening for COVID-19: a Proposed Cutoff Score for the Greek Version of the Fear of COVID-19 Scale (FCV-19S)", International Journal of Mental Health and Addiction, 2020<br>Publication	7%
2	www.ncbi.nlm.nih.gov<br>Internet Source	4%
3	www.researchsquare.com<br>Internet Source	3%
4	Fatema Mohsen, Batoul Bakkar, Judy Kikhia, Dana Mchantaf, Samar Aldakkak, Alma Najjar, Marah Marrawi, Youssef Latifeh. "Psychological Impacts and Attitudes of the Syrian Population Regarding the COVID-19 Pandemic and Face Masks During the First Syrian Lockdown", Research Square Platform LLC, 2021<br>Publication	2%

clinicaltrials.gov

5	Internet Source	2%
6	Batoul Bakkar, Fatema Mohsen, Humam Armashi, Marah Marrawi, Nizar Aldaher. "A Crisis within a Crisis: An Assessment of COVID-19 Attitude and Practice among Syrians - A Cross-sectional Study", Research Square, 2021<br>Publication	1%
7	link.springer.com<br>Internet Source	1%
8	www.jmir.org<br>Internet Source	1%
9	www.tandfonline.com<br>Internet Source	1%
10	www.grin.com<br>Internet Source	1%
11	Amar Prashad Chaudhary, Narayan Sah Sonar, Jamuna TR, Moumita Banerjee, Shailesh Yadav. "Impact on Mental Health of students due to restriction caused by COVID-19 pandemic: Cross-sectional study", Cold Spring Harbor Laboratory, 2021<br>Publication	1%
12	www.frontiersin.org<br>Internet Source	1%

13	Fatema Mohsen, Batoul Bakkar, Sara Melhem, Roula Altom, Bisher Sawaf, Imad Alkhija, Louei Darjazini Nahas. "Foreign body aspiration in a tertiary Syrian centre: A 7-year retrospective study", Heliyon, 2021<br>Publication	1 %
14	ikee.lib.auth.gr<br>Internet Source	1 %
15	Fatema Mohsen, Mosa Shibani, Nazir Ibrahim, Ghaith Alhourani et al. "Knowledge, Attitude, and Practice Regarding HIV, HBV, and HCV Among Medical Students of Syrian Private University, Damascus, Syria", International Quarterly of Community Health Education, 2021<br>Publication	1 %
16	opendevelopmentcambodia.net<br>Internet Source	<1 %
17	Rubia Carla Formighieri Giordani, Milene Zanoni da Silva, Camila Muhl, Suely Ruiz Giolo. "Fear of COVID-19 scale: Assessing fear of the coronavirus pandemic in Brazil", Journal of Health Psychology, 2020<br>Publication	<1 %
18	Submitted to University of Edinburgh<br>Student Paper	<1 %
	citeseerx.ist.psu.edu	

19	Internet Source	<1 %
20	www.termedia.pl<br>Internet Source	<1 %
21	Submitted to Al Balqa Applied University<br>Student Paper	<1 %
22	Sa’ed H Zyoud, Samah W Al-Jabi, Waleed M Sweileh, Masa M Nabulsi, Mais F Tubaila, Rahmat Awang, Ansam F Sawalha. "Beliefs and practices regarding childhood fever among parents: a cross-sectional study from Palestine", BMC Pediatrics, 2013<br>Publication	<1 %
23	Submitted to colorado-technical-university<br>Student Paper	<1 %
24	Submitted to University of East London<br>Student Paper	<1 %
25	W. Elshami, T.N. Akudjedu, M. Abuzaid, L.R. David, H.O. Tekin, B. Cavli, B. Issa. "The radiology workforce's response to the COVID-19 pandemic in the Middle East, North Africa and India", Radiography, 2020<br>Publication	<1 %
26	international-review.icrc.org<br>Internet Source	<1 %

27	Danielle Saadeh, Aline Hajj, Diana Malaeb, Hala Sacre, Chadia Haddad, Pascale Salameh. "Assessing Changes in Lifestyle and Health-Related Behaviors During the COVID-19 Pandemic: A Cross-Sectional Web-Based Survey Among Lebanese Adults", Research Square Platform LLC, 2021<br>Publication	<1 %
28	David Gunnell, Louis Appleby, Ella Arensman, Keith Hawton et al. "Suicide risk and prevention during the COVID-19 pandemic", The Lancet Psychiatry, 2020<br>Publication	<1 %
29	Submitted to University of Western Australia<br>Student Paper	<1 %
30	www.mdpi.com<br>Internet Source	<1 %
31	www.wieismijn.net<br>Internet Source	<1 %
32	Petros A Galanis, Emmanouela Petrogianni, Irene Vraka, Olympia Konstantakopoulou et al. "Fear of COVID-19 among nurses in mobile COVID-19 testing units in Greece", Cold Spring Harbor Laboratory, 2021<br>Publication	<1 %
33	Akihiro Masuyama, Hiroki Shinkawa, Takahiro Kubo. "Validation and Psychometric	<1 %

Properties of the Japanese Version of the Fear of COVID-19 Scale Among Adolescents", International Journal of Mental Health and Addiction, 2020

Publication

34	Chung-Ying Lin, Anders Broström, Mark D. Griffiths, Amir H. Pakpour. "Investigating mediated effects of fear of COVID-19 and COVID-19 misunderstanding in the association between problematic social media use, psychological distress, and insomnia", Internet Interventions, 2020<br>Publication	<1 %
35	Chung‐Ying Lin, Wen‐Li Hou, Mohammed A. Mamun, José Aparecido da Silva et al. "Fear of COVID‐19 Scale (FCV‐19S) across countries: Measurement invariance issues", Nursing Open, 2021<br>Publication	<1 %
36	Cuneyt Evren, Bilge Evren, Ercan Dalbudak, Merve Topcu, Nilay Kutlu. "Measuring anxiety related to COVID-19: A Turkish validation study of the Coronavirus Anxiety Scale", Death Studies, 2020<br>Publication	<1 %
37	Hanan F. Hussein, Eglal H. Al-Gohary, Amal G. Mohamed, Amal A. A. Abd El-Salam. "Evaluation of Red Cell Distribution Width and	<1 %

Platelet Indices in Children with Chronic Heart Disease", Open Journal of Pediatrics, 2021
Publication

38	Xinli Chi, Shiyun Chen, Yuying Chen, Danying Chen et al. "Psychometric Evaluation of the Fear of COVID-19 Scale Among Chinese Population", International Journal of Mental Health and Addiction, 2021<br>Publication	<1 %
39	journals.plos.org<br>Internet Source	<1 %
40	software-ab.informatik.uni-tuebingen.de<br>Internet Source	<1 %
41	Marwa Summaka, Hiba Zein, Ibrahim Naim, Sleiman Fneish. "Assessing the psychological impact of COVID-19 outbreak and its related factors on Lebanese individuals with physical disabilities", Disability and Health Journal, 2021<br>Publication	<1 %
42	Zeynep Şimşir, Hayri Koç, Tolga Seki, Mark D. Griffiths. "The relationship between fear of COVID-19 and mental health problems: A meta-analysis", Death Studies, 2021<br>Publication	<1 %
43	abfer.org<br>Internet Source	<1 %

44 pediatrics.aappublications.org <1 %
Internet Source

45 Najmuj Sakib, A. K. M. Israfil Bhuiyan, Sahadat Hossain, Firoj Al Mamun et al. "Psychometric Validation of the Bangla Fear of COVID-19 Scale: Confirmatory Factor Analysis and Rasch Analysis", International Journal of Mental Health and Addiction, 2020 <1 %
Publication

46 Sevinc Kulekcioglu, Merve Akyüz, Özenç İnan, Alp Çetin. "Coronaphobia in Patients With Fibromyalgia.", Research Square, 2020 <1 %
Publication

47 Yasmin Al-Shannaq, Anas A. Mohammad, Yousef Khader. "Psychometric Properties of the Arabic Version of the Fear of COVID-19 Scale (FCV-19S) Among Jordanian Adults", International Journal of Mental Health and Addiction, 2021 <1 %
Publication

48 Eccleston, Christopher, Emma Fisher, Lorraine Craig, Geoffrey B Duggan, Benjamin A Rosser, Edmund Keogh, and Christopher Eccleston. "Psychological therapies (Internet-delivered) for the management of chronic pain in adults", Cochrane Database of Systematic Reviews, 2014. <1 %
Publication

49	Ismaël Conejero, Sofian Berrouiguet, Deborah Ducasse, Marion Leboyer, Vincent Jardon, Emilie Olié, Philippe Courtet. "Épidémie de COVID-19 et prise en charge des conduites suicidaires: challenge et perspectives", L'Encéphale, 2020<br>Publication	<1 %

Exclude quotes	On	Exclude matches	Off
Exclude bibliography	On		
