# Supplementary material for: Fear among Syrians: A Proposed Cutoff Score for the Arabic Fear of COVID-19 Scale
Source: PLoS One. 2022 Mar 11;17(3):e0264257. doi: 10.1371/journal.pone.0264257 (PMC8916619; doi:10.1371/journal.pone.0264257)
Supplement: Attachment — Submitted filename: Response Letter #4.docx [file pone.0264257.s005.docx]

Response letter

**We would like to thank both the editor and reviewers for spending their valuable time reviewing this manuscript. We have thoroughly assessed the comments and implemented them into our revised manuscript, now that this article has been made suitable, we aspire to have this article published in your journal.**

**The following letter will report the responses to the reviewers’ comments.**

We've checked your submission and before we can proceed, we need you to address the following issues:

Author response: We will address the following issue provided.

2.Please include a separate legend for each figure in your manuscript.

Author response: We have included figure captions in the manuscript text in read order, immediately following the paragraph where the figure is first cited.
