# Supplementary material for: Fear among Syrians: A Proposed Cutoff Score for the Arabic Fear of COVID-19 Scale
Source: PLoS One. 2022 Mar 11;17(3):e0264257. doi: 10.1371/journal.pone.0264257 (PMC8916619; doi:10.1371/journal.pone.0264257)
Supplement: Attachment — Submitted filename: Response Letter #5.docx [file pone.0264257.s006.docx]

Response letter

**We would like to thank both the editor and reviewers for spending their valuable time reviewing this manuscript. We have thoroughly assessed the comments and implemented them into our revised manuscript, now that this article has been made suitable, we aspire to have this article published in your journal.**

**The following letter will report the responses to the reviewers’ comments.**

1. Please report the average variance extracted (AVE) scores and items’ outer loadings as an assessment of construct validity for all of the measures used in the study. Also, computing the composite reliability (CR) would be great.

Author response: We have added a table that includes factor loading, corrected item total correlation, cronbach’s alpha, inter item correlation range, skewness, kurtosis, and Kaiser meyer-olkin for each item of the FCV-19S. The data is presented in table 3. We also calculated the AVE and CR and reported them in the results section.

2. The script does not describe how the discriminant validity was assessed (e.g., HTMT). Please, revise accordingly.

Author response: We have reported the discriminant validity.

3. Given the ongoing turmoil in Syria, literature on “Wartime Crisis Perceptions” should be brought up in the discussion, mainly concerning not considering this variable in the study, hence offering directions for future research. Similarly, COVID-19 perception (defined as “as the perceived probability of discomfort and/or worry, during COVID-19 pandemic, concerning the pandemic adverse health, economic and social ramifications articulated as disruptions to the people’s pre-pandemic everyday life – lead to redefining of the everyday life to the new normal.”) which conceptually should occur before developing fears and other negative experiences is warranted a discussion here too.

Author response: A very important point, we have included this point in the limitations section. Highlighting the need for this point to be further assessed.

4. Ensure the accuracy of terms used in the paper (e.g., anxiety, fear are emotions that our perception of events would trigger).

Author response: We have revised the manuscript for the point mentioned above.

5. The whole manuscript would benefit from proper proofreading.

Author response: We have revised the manuscript.

6. I found some similarity issues that you will need to remedy. Please, see attached the similarity check report and paraphrase/cite accordingly.

Author response: We have rephrased as many sentences as we could from the file sent. However, there are many phrases and words we cannot change, for example “fear of COVID-19 scale” we noticed these were picked up. Also, places we can not change include the affiliation section. We hope the manuscript is better now.

7. Finally, given the study context, an Arabic abstract of the research, as supplementary material, would add more visibility to your paper. Also, ensure a list of the translated items is offered in Arabic as supplementary material too.

Author response: We have translated the abstract into Arabic and have attached it as a supplementary file. The items have been translated into Arabic and are available in the supplementary material as S3.
